# Supplementary material for: State-transition modeling of blood transcriptome predicts disease evolution and treatment response in chronic myeloid leukemia
Source: Leukemia. 2024 Feb 2;38(4):769–80. doi: 10.1038/s41375-024-02142-9 (PMC10997512; doi:10.1038/s41375-024-02142-9)
Supplement: Supplementary file 1 — Supplemental Figures [file 41375_2024_2142_MOESM1_ESM.docx]

Supplementary Figures for:

**State-transition Modeling of Blood Transcriptome Predicts Disease Evolution and Treatment Response in Chronic Myeloid Leukemia**

David E. Frankhouser^1^*^, Russell C. Rockne^1^*^, Lisa Uechi^1^, Dandan Zhao^2^, Sergio Branciamore^1^, Denis O’Meally^3^, Jihyun Irizarry^2^, Lucy Ghoda^2^, Haris Ali^2^, Jeffery M. Trent^4^, Stephen Forman^2^, Yu-Hsuan Fu^2^, Ya-Huei Kuo^2^, Bin Zhang^2#*^, Guido Marcucci^2#*^

*Corresponding authors.

David Frankhouser ([dfrankhouser@coh.org](mailto:dfrankhouser@coh.org))

Russell Rockne ([rrockne@coh.org](mailto:rrockne@coh.org))

Guido Marcucci ([gmarcucci@coh.org](mailto:gmarcucci@coh.org))

Bin Zhang ([bzhang@coh.org](http://bzhang@coh.org))

Contents:

Supplementary Figures S1-S8

Legends for Table S1-S4

**Figure S1: Features of the CML state-space.**

**A)** The CML state-space was correlated with clinical markers of CML the myeloid population in the peripheral blood (*left*) and the log-expression of BCR::ABL (*right*). Linear fits (*black dashed*) were used to show that the CML state-space had the best correlation with both myeloid and BCR::ABL (Table S1). **B)** The percent of variation encoded by each PC that resulted from the SVD operation was plotted and showed that the variation encoded by PCs decreased and leveled off after PC2

**Figure S2: Mean squared displacement estimates for the diffusion coefficients.**

**A)** A mean square displacement (MSD) was performed for each mouse using their trajectories in the CML state-space as a function of time. **B)** For each cohort of mice, a linear fit of all trajectories in the cohort was calculated and the slope of the line was used to estimate the diffusion coefficient ($\beta$). Treatment cohorts were shown only until treatment was initiated.

**Figure S3: Analysis of CML disease states.**

**A)** Differentially expressed genes (DEGs) were identified between all pairwise comparisons of the CML disease states (Es, Ts, Ls) and healthy controls (Hs). The results for the intersection between each comparison were plotted separately for all genes (*top*), the upregulated genes in each comparison (*left*), and the downregulated genes in each comparison (*right*). **B)** Gene set enrichment analysis (GSEA) was also performed on all comparisons using the Hallmark gene sets. Here, the inter-CML disease state comparisons (pairwise comparisons of Es, Ts, and Ls) were plotted using the normalized enrichment score (NES) to show the direction of the expression in all significantly enriched gene sets (adjusted p-value < 0.001). **C)** The DEGs unique to each disease state were defined by making intersections between the relevant comparisons to determine which gene expression changes only occurred in each disease state. **D)** The percent of myeloid cells in the peripheral blood was compared in each disease state using a t-test (*left*). The myeloid growth rate was calculated as the derivative of the spline fits for each mouse’s myeloid percentage (*right*) and a t-test was again used to compare growth rate between disease states (significant p-value < 0.05).

**Figure S4: Eigengenes quantify CML contribution of genes and groups of genes.**

**A)** Using the eigengenes, which were defined as the CML state-space (PC2) loading values, we determined a gene’s quantitative contribution to CML state-transition. For each gene, the CML contribution was determined by combining the eigengene with the observed gene expression change using the table (*left*) to determine whether the contribution was pro- or anti-CML. The CML contribution was illustrated for a set of genes (*right*) by plotting the CML contribution vs the PC1 value for all genes in the set. The total contribution of the set was represented by the mean vector of all genes in the set (*black arrow*). **B)** The CML contribution was used to summarize the overall effect of each DEG comparison on CML state-transition. **C)** The CML contribution was summarized for the three inter-CML comparison’s GSEA results by plotting the total CML contribution for the genes in leading edge of each significantly enriched gene set (adjusted p-value < 0.0001). Missing bars represent gene sets that were not significant in that comparison.

**Figure S5: Expression dynamics of the unique disease-state DEGs.**

A correlation analysis using all mice from the CML cohort was used to determine which genes have similar expression dynamics over CML development. Using hierarchical clustering on the correlation matrix, gene modules were defined as a cluster of genes with a mean correlation coefficient greater than 0.25. To visualize how the expression changed over CML, the average expression of each gene module was plotted for each sample as a function of the CML state-space of the sample and summarized using a loess fit (*red line*). Finally, the overall CML contribution (*black arrow*) of each gene module was determined. This process was performed on the **A)** DEGs unique to Es, **B)** the DEGs unique to Ts, and **C)** the DEGs unique to Ls.

**Figure S6: Driver gene of CML state-transition.**

**A)** Process for identifying the T-Es and the T-Ls drive genes. **B)** Venn diagram of the T-Es and T-Ls driver genes. **C)** Protein-protein interaction networks for the T-Es (*left*) and the T-Ls (*right*) driver genes. STRINGdb was used to identify high confidence interactions (interaction score > 900). Each gene is colored based on the observed log foldchange between Ts vs Hs for the T-Es driver genes or Ls vs Hs for the T-Ls driver genes. **D)** To further refine the processes involved in the large network of the T-Ls driver genes, three subnetworks were identified, and the top five most significantly enriched Hallmark gene sets for each cluster were reported to characterize each cluster (network interaction p-values < 0.0001).

**Figure S7: Analysis of treatment cohorts.**

**A)** Clinical variables plotted vs time for Tet-on Tet-off (TOTO) mice (*top*) and TKI mice (*bottom*). **B)** Intersection up- and down-regulated treatment cohorts with the disease states (Es, Ts, and Ls) compared with the control (Hs). **C)** To compare two measures of transcriptomic similarity between two groups of samples, the number of DEGs was compared to the average distance between the samples of each group in the CML state-space for all DEG comparisons. **D)** The CML contribution of the DEG (*left*) and the significantly enriched Hallmark gene sets from GSEA (adjusted p-value < 0.001; *right*) that resulted from the TOTO post-Rx samples vs healthy control (Hs) samples. **E)** An analysis comparing the early vs late control CML samples due to the observed change in CML state-space location over the course of the experiment (*top*). The total CML contribution was calculated for all DEGs that resulted from comparing the early vs late control samples (*bottom left*) which showed a small pro-CML effect in the late control mice. GSEA was also performed, and all significantly enriched Hallmark gene set pathways were upregulated in the late control mice (adjusted p-value < 0.001; *bottom right*). **F)** Total CML contribution for all significantly enriched Hallmark gene sets after GSEA for the TKI Rx and TKI post-Rx samples when compared to controls (adjusted p-value < 0.001). Missing bars indicate a gene set that was not significant in that comparison. **G)** GSEA results from the TKI post-Rx samples compared to the Ls CML samples. Significantly enriched Hallmark gene sets were shown with enrichment in expression with respect to the TKI post-Rx samples (*top*). The total CML contribution of the TKI post-Rx samples was summarized for each gene set (*bottom*).

**Figure S8: Treatment state-transition probability**

The solution for the state-transition model is shown for TOTO (*left*) and TKI (*right*) treatments as a probability density over time which represents the likelihood of finding a sample in the CML state-space as time evolves.

**Table S1:** Correlation analysis of BCR::ABL expression and myeloid population with each principal component.

**Table S2:** DEG and GSEA results for both CML disease state comparisons (Es, Ts, Ls) vs healthy controls (Hs) and inter-CML disease state comparisons.

**Table S3:** Hallmark gene set enrichment analysis for state-transition driver genes at T-Es and T-Ls.

**Table S4:** DEG and GSEA results for treatment groups (TOTO post-Rx, TKI Rx, TKI post-Rx) compared to both CML disease states (Es, Ts, Ls) and healthy controls (Hs)

**Table S5**: Values of parameters used in the modeling.

|  | $\boldsymbol{\gamma}$  (*x/time*) | $\boldsymbol{\beta}$  (*x^2^/time*) | $\boldsymbol{\lambda}$ | $\boldsymbol{a}$  (*A.U.*) | $\boldsymbol{c}_{\mathbf{1}}$  (*A.U.*) | $\boldsymbol{c}_{\mathbf{2}}$  (*A.U.*) | $\boldsymbol{c}_{\mathbf{3}}$  (*A.U.*) | $\boldsymbol{c}_{\mathbf{4}}$  (*A.U.*) | $\boldsymbol{c}_{\mathbf{5}}$  (*A.U.*) |
| --- | --- | --- | --- | --- | --- | --- | --- | --- | --- |
| TOTO | $\gamma_{TOTO}=$1.1 | $\beta_{Tet}^{-1}=$0.088 $\beta_{Tet-on}^{-1}=$0.001 |  | 1 | 1 | 0.9231 | 0.4744 | 0.2393 | 0 |
| TKI | $\gamma_{TKI}=$1.3 | $\beta_{TKI}^{-1}=$0.037 $\beta_{TKI-on=}^{-1}$0.001 $\beta_{TKI-off}^{-1}=$0.001 | 6.9315 | 1 | 1 | 0.9231 | 0.4744 | 0.2393 | 0 |
| CML |  | $\beta_{CML}^{-1}$=0.005 |  | 100 | 1 | 0.9231 | 0.4744 | 0.2393 | 0 |
